# The Good, the Bad, and the Ugly of Pregnancy Nutrients and Developmental Programming of Adult Disease

**DOI:** 10.3390/nu11040894

**Published:** 2019-04-20

**Authors:** Chien-Ning Hsu, You-Lin Tain

**Affiliations:** 1 Department of Pharmacy, Kaohsiung Chang Gung Memorial Hospital, Kaohsiung 833, Taiwan; chien_ning_hsu@hotmail.com; 2 School of Pharmacy, Kaohsiung Medical University, Kaohsiung 807, Taiwan; 3 Department of Pediatrics, Kaohsiung Chang Gung Memorial Hospital and Chang Gung University College of Medicine, Kaohsiung 833, Taiwan; 4 Institute for Translational Research in Biomedicine, Kaohsiung Chang Gung Memorial Hospital and Chang Gung University College of Medicine, Kaohsiung 833, Taiwan

**Keywords:** developmental origins of health and disease (DOHaD), gut microbiota, non-communicable disease, nutrient-sensing signal, nutrition, oxidative stress, pregnancy, reprogramming

## Abstract

Maternal nutrition plays a decisive role in developmental programming of many non-communicable diseases (NCDs). A variety of nutritional insults during gestation can cause programming and contribute to the development of adult-onset diseases. Nutritional interventions during pregnancy may serve as reprogramming strategies to reverse programming processes and prevent NCDs. In this review, firstly we summarize epidemiological evidence for nutritional programming of human disease. It will also discuss evidence from animal models, for the common mechanisms underlying nutritional programming, and potential nutritional interventions used as reprogramming strategies.

## 1. Introduction

Maternal nutrition plays an essential role in fetal growth and development. It has long been known that adverse nutritional conditions during pregnancy may permanently change the structure and function of specific organs in the offspring, leading to many adult diseases. This concept is now currently referred to as the Developmental Origins of Health and Disease (DOHaD) [1]. Conversely, the DOHaD concept provides a strategy to reverse programming processes by shifting the therapeutic interventions from adulthood to fetal or infantile stage, before clinical phenotype becomes evident [2,3]. Nutritional interventions during pregnancy have started to gain importance as a reprogramming strategy to prevent DOHaD-associated diseases [4,5,6].

Nutritional exposure at early life is particularly important, as the plasticity of developing organs that shape the way in which the body reacts to challenges in later life. This review highlights evidence for the impact of nutritional programming on offspring health and the role of nutritional interventions as a reprogramming strategy in the emerging area of DOHaD research.

## 2. Nutritional Programming of Health and Disease: Good, Bad, or Ugly?

The term nutritional programming describes the process through which exposure to early-life nutritional stimuli brings about morphological changes or functional adaption of the offspring [7,8]. The long-term consequences of nutritional insults are variable and depend on several factors such as the type of nutrient, exposure duration and intensity, species, sex, and the critical time-windows of development during which it is applied. Nutritional programming is emerging as a critical risk factor for a number of non-communicable diseases (NCDs), including hypertension, cardiovascular disease, diabetes, obesity, allergic diseases, kidney disease, neurocognitive impairments, nonalcoholic fatty liver disease (NAFLD), and metabolic syndrome [3,4,5,6,7,8,9,10]. NCDs constitute the main cause of death all over the world. Although NCDs are generally preventable, current approaches are obviously insufficient.

Conversely, nutritional programming can also be advantageous. From an evolutionary perspective, developmental plasticity seems to represent an adaptive process. Developmental plasticity is beneficial for fitness, despite such benefits may come at a cost to health outcomes. Developmental plasticity can offer a survival advantage to the offspring based on evolutionary grounding [11]. Also, developmental plasticity is beneficial when environmental conditions change within generations [11]. Importantly, several reports suggest, at least in animal models, that developmental programming of adult disease is potentially reversible by nutritional interventions during the period of developmental plasticity. In genetic models of hypertension, early-life nutritional interventions can improve cardiovascular outcomes in adult offspring [12,13]. The identification of critical time-windows and specific nutritional interventions is thus a promising way to explore in order to offer novel reprogramming strategies.

Considering the good and bad sides of nutritional programming during pregnancy, there is still an ugly side of it. Currently, little information exists on certain nutrients for women with pre-existing deficiencies, as the dietary reference intakes are established for healthy individuals. Almost all dietary reference intake reports lack long-term offspring outcome data to accurately inform recommendations for women in the pregnancy stage. However, currently in human studies it is difficult to confirm causation linking maternal nutrition status with phenotypes in offspring. Also, these cohorts do not identify molecular mechanisms by which the phenotype is generated and aid in developing specific nutritional interventions for a wide spectrum of adult diseases. As a consequence of ethical considerations concerning what is achievable or not in human studies, animal models are essential. It is for this reason that much of our knowledge of the type of nutritional insults driving the programming process, the critical time-window of vulnerability for nutritional insults, potential mechanisms underpinning nutritional programming, and reprogramming strategy largely come from studies in animal models.

On the contrary, there are some limitations of animal models when translating into replications in human trials. Possible problems include different models with varying similarity to the human conditions, variability in animals for study, follow-up duration may not correspond to disease latency in humans, and outcome measures have uncertain relevance to the human conditions [14].

Despite interesting results using nutritional interventions to prevent various adult diseases have been obtained from animal models, many challenges still lie ahead for successful translation of promising animal therapies to humans. As nutrients do not drive their programming effect independently from each other, key questions for future research include what are the nutrient–nutrient interactions, nutrient–drug interactions, and nutrient–environment interactions that can affect the programming power on pregnancy and offspring outcomes.

**Figure 1:**
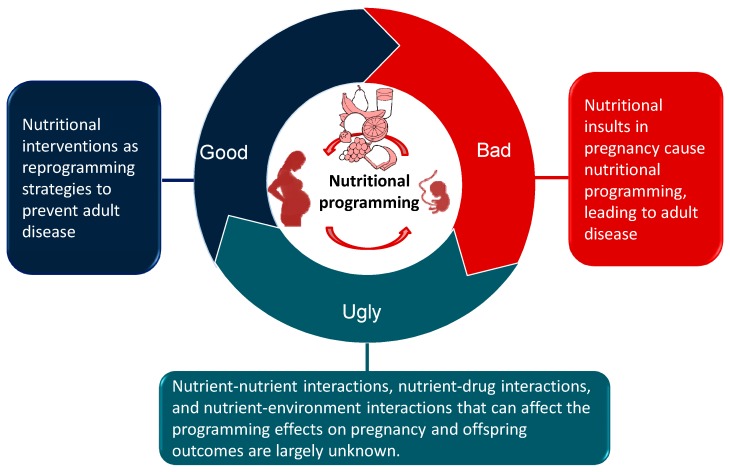
Schematic illustration of the good, the bad, and the ugly sides of nutritional programming involved in developmental programming of health and disease.

A schematic summarizing the good, the bad, and the ugly sides of nutritional programming involved in the developmental programming of adult diseases is presented in Figure 1.

## 3. Epidemiological Evidence for Nutritional Programming of Human Disease

Excessive or insufficient consumption of a specific nutrient has been linked to developmental programming of a variety of NCDs [15,16,17,18,19,20,21,22,23,24,25,26,27]. Table 1 mainly summarizes cohort studies documenting adverse offspring outcomes in response to both undernutrition and overnutrition during pregnancy [15,16,17,18,19,20,21,22,23,24,25,26,27]. Although low birth weight is considered as a surrogate marker of early-life nutrition and numerous studies of maternal under- and over-nutrition have revealed its association with offspring health [28], we have restricted this review only to adverse outcomes starting from childhood.

First, several famine cohort studies consistently showed that offspring exposed to gestational famine are more vulnerable to metabolic syndrome-related disorders [15,16,17,18]. One famous example is the Dutch famine study, which demonstrated that undernutrition in pregnancy is associated with increased risk of adult offspring developing coronary heart disease, hyperlipidemia, obesity, obstructive airways disease, kidney disease, and hypertension [16,18]. The Dutch famine study also showed that offspring exposed to famine in early gestation are prone to developing coronary heart disease, hyperlipidemia, and obesity. Exposure in mid gestation is associated with obstructive airways disease and microalbuminuria. These findings suggest that the timing of undernutrition in pregnancy predicts which organ system is reprogrammed and leads to the development of adult diseases [16]. However, no such consequence was observed in the Leningrad siege famine study [29]. The dissimilar effects of exposure to the two famines suggest the importance of the timing during gestation and the organs and systems developing during that critical time-window. A recent meta-analysis of 20 studies reported that fetal famine exposure may increase the risk of overweight and obesity [30].

Overnutrition is a form of malnutrition in which the intake of nutrients or a specific nutrient is oversupplied, especially in unbalanced proportions. Only a small number of studies have examined the contribution of excessive intake of a specific macronutrient during pregnancy to the adverse offspring outcomes [23,24,25,26,27]. It has been demonstrated that high-free-sugar intake in pregnancy is associated with childhood atopy and asthma [23]. There is also an association between high-protein intake in pregnancy and the risk for high blood pressure (BP) in adult offspring [24,25,26]. Additionally, consumption of a high-fat diet during pregnancy has been reported to increase the risk of overweight in male adult offspring [27]. It is noteworthy that most studies mainly focused on cardiovascular outcomes and metabolic outcomes, rather less attention has been paid to other programming effects, including neurobehavioral, endocrine, and respiratory outcomes.

Since epidemiological studies do not dissect the physiological and molecular mechanisms by which the disorder is created, animal models with full control over the dietary manipulations are essential in the discovery of mechanisms underlying nutritional programming and the development of ideal nutritional interventions before they are implemented in humans.

## 4. Insights from Animal Models of Nutritional Programming

### 4.1. Animal Models for Nutitional Programming

Animal models particularly aid in inferring relevant information for possible translation to clinical practice in the field of nutritional programming. Using animal model with proven construct validity can increase the probability of successful translation of preclinical findings to clinical application. The translatability may further be increased by using a broader spectrum of relevant animal models. The choice of one animal model over another mainly depends on its similarity to the human diseases. For example, rabbits offer a better model of human atherosclerosis than rodents as they are prone to develop fatty streak lesions in studies of high-fat intake [31]. Although non-human primate is considered as the gold standard because of their similarity to humans, the most common species used are rodents in the DOHaD field [32]. Other species such as rabbits, sheep, pigs have been used depending on the experimental approaches, to study nutritional programming related to offspring outcomes [32]. Mice models provide a low-cost and easy-to-handle option, allowing genetic modification. Rabbits are suitable for studies on the blastocyst due to their large size. Additionally, their placenta structure and lipid metabolism are close to those in humans. Sheep are a monotocous species with a long gestational period as in humans. Pigs are considered as a good model for studying early stages of fertilization and development. Thus, animal studies play a pivotal role for us to clarify programming effects, identify critical developmental windows, and develop ideal reprogramming strategies for nutritional programming. The range of nutritional insults that have been utilized can be grouped into models that seek to restrict calorie intake, manipulate macronutrient intake, or restrict micronutrient intake.

### 4.2. Animal Models of Maternal Caloric rRstriction

Rodent studies of calorie restriction ranging from 30–70% to pregnant dams showed hypertension in the adult offspring [33,34,35]. Offspring that experienced a 50% calorie restriction during the last week of gestation showed impairment of beta cell development [36]. In rodents, severe 70% caloric restriction during pregnancy resulted in obesity, hypertension, hyperleptinemia, and hyperinsulinism in adult offspring [37]. Similar adverse cardiovascular outcomes have been observed in cows and sheep [38,39]. Pups exposed to a more severe degree of caloric restriction were likely to develop hypertension earlier [5]. Additionally, the severity of adverse offspring outcome seems relevant to the timing of exposure. In a maternal 50% caloric restriction sheep model, no major change in glucose–insulin homeostasis was observed in adult offspring born of undernourished ewes during early pregnancy [40]. However, maternal undernutrition during late pregnancy caused glucose intolerance and insulin resistance in adult offspring. These findings suggest that undernutrition in late pregnancy, during the period of maximal fetal growth, impacts negatively on the subsequent adult offspring’s glucose–insulin homeostasis.

### 4.3. Animal Models of Macronutrients Imbalance

Macronutrients include carbohydrates, proteins, and fats. Sugar consumption, particularly fructose, has grown over the past several decades and its growth has been paralleled by an increase in obesity, diabetes, and hypertension [41]. Several rodent studies showed that maternal diets consisting of 75% simple carbohydrates (dextrose and maltodextrin) led to a significantly higher body weight gain in offspring [42,43,44]. Fructose is a monosaccharide naturally present in fruits. However, most of the increase in fructose consumption now is derived from refined sugars and high-fructose corn syrup [45]. As we reviewed elsewhere [46], consumption of high-fructose alone or as a part of diet by rodent mothers induces several features of metabolic syndrome in adult offspring, including hypertension, insulin resistance, obesity, hepatic steatosis, and dyslipidemia.

Of note, adverse effects of fructose feeding depend on the amount and duration of fructose consumption [47]. Despite being viewed as far in excess of a relevant load, most rodent studies have been performed using diets containing 50–60% fructose [48,49]. However, a recent study indicated that maternal consumption of 10% w/v fructose significantly increased BP in mice offspring after 1 year [50]. On the other hand, several studies used fructose as a part of maternal diet along with fat and salt to induce hypertension in adult offspring [51,52]. As Western diet is characterized by the intake of high-sugar drinks, high-fat products, and excess salt, it is important to dissect the interplay between fructose, fat, and salt on the nutritional programming. Indeed, animal studies examining the combined effects of key components of the Western diet have shown their synergistic effects of fructose, fat, and salt on the elevation of BP in adult offspring [53,54].

Protein is another macronutrient. The low-protein model has been extensively used to study the mechanisms of nutritional programming. In rodents, protein restriction during pregnancy leads to intrauterine growth retardation (IUGR) with subsequent hyperglycemia, glucose intolerance, insulin resistance, obesity, and adipocyte hypertrophy [10]. Additionally, rodent studies of protein restriction ranging from 6–9% to pregnant dams showed hypertension in the adult offspring [55,56,57,58,59]. Similar to caloric restriction, a greater degree of protein restriction shows a higher propensity to develop hypertension earlier in offspring [5]. Similar observations in several model species have confirmed the effects of maternal low-protein diet on the above-mentioned metabolic syndrome-related phenotypes in mammals [59]. Programming effects of protein restriction occur not only during pregnancy but also through breastfeeding. Mice offspring born to dams fed with low-protein diet during lactation have impaired cardiac structure and function that limit exercise/functional capacity [60].

Maternal high-fat diet is a widely used animal model to induce developmental programming of obesity and related disorders [61]. Adult offspring exposed to a maternal high-fat consumption in utero developed insulin resistance, increased lipid profile, hepatic steatosis, increased visceral fat mass, and adipocyte hypertrophy [61,62,63]. Nevertheless, maternal high-fat-diet-induced responses of BP include an increase [64,65] or no change [66,67], mainly depending on strain, sex, age, and diverse fatty acid compositions.

Additionally, alteration of the protein-to-carbohydrate ratio of the maternal diet was related to an adverse outcome in offspring. Male offspring rats born of dams exposed to a diet with high-protein/carbohydrate ratio were characterized by elevated BP and a greater degree of glomerulosclerosis [68]. These data are in agreement with human studies showing that a maternal high-protein, low-carbohydrate diet is linked to elevated BP in the offspring [24,25].

### 4.4. Animal Models of Micronutrients Deficiency

There is also evidence to support that deficiencies in micronutrients, including minerals, trace elements and vitamins, in pregnant mothers are associated with the development of NCDs. Evidence in rats showed both maternal low- and high-salt diets lead to elevated BP in male adult offspring [69]. Additionally, maternal calcium-deficient diet increased BP and insulin resistance in adult rat offspring [70,71]. Furthermore, magnesium-deficient diet in pregnancy caused impaired kidney function but had no effect on BP in rat offspring [72]. Deficiencies in trace elements and vitamins in pregnant mothers are associated with the development of hypertension, impaired glucose tolerance, and insulin resistance in their adult offspring [73,74,75,76,77,78]. These micronutrients include iron [73,74], zinc [75,76], and vitamin D [77,78]. Additionally, maternal folate and vitamin B12 restriction increased visceral adiposity and altered lipid metabolism in rat offspring [79].

The vitamins folic acid, B12, B6, and B2 have a key role as coenzymes in one-carbon metabolism, serving as methyl-donor nutrients. Because one-carbon metabolism is required for epigenetic regulation of gene expression, pregnant women are recommended to eat methyl-donor food to reduce adverse birth outcomes [80]. These foods contain nutrients that directly provide methyl-donors or serve as cofactors, such as methionine, folic acid, vitamins B2, B6, B12, and choline. Although methyl-donor supplementation may be of benefit in preventing disease, we recently found that pregnant rats fed with high-methyl-donor diet led to hypertension in male adult offspring [81]. Thus, additional studies should be taken to assess the programming effects of these methyl-donor nutrients and to solve the current conflicting data in regards to maternal methyl-donor diet.

Vitamin C, E, B6, and coenzyme Q-10 have shown beneficial effects on lowering BP [82]. However, whether deficiencies of these micronutrients on pregnant mothers lead to programmed hypertension in their offspring remains unclear. So far, no studies have been conducted examining the role of deficient non-essential nutrients on developmental programming of adult disease.

## 5. Common Mechanisms Underlying Nutritional Programming of Disease

In view of the fact that diversely nutritional insults in gestation create very similar outcome in adult offspring, there might be some common mechanisms contributing to the pathogenesis of nutritional programming of disease. Currently, possible mechanisms underlying nutritional programming include nutrient-sensing signals, oxidative stress, tissue remodeling, epigenetic regulation, gut microbiota, and sex differences [5,6,7,8,9,10,59]. Each mechanism will be discussed in turn.

### 5.1. Nutrient-Sensing Signals

Fetal metabolism and development are determined by maternal nutritional status via nutrient-sensing signals. Sirtuin 1 (SIRT1), cyclic adenosine monophosphate (AMP)-activated protein kinase (AMPK), mammalian target of rapamycin (mTOR), and peroxisome proliferator-activated receptors (PPARs) are well-known nutrient-sensing signals [83]. In excessive nutrient conditions, the mTOR is activated by increases in glucose, amino acid, and insulin levels, while in nutrient-depleted conditions, AMPK and SIRT1 are activated by increases in intracellular AMP and NAD^+^ levels, respectively. Dysregulated nutrient-sensing signals are present in several metabolic diseases [83]. The interplay between AMPK and SIRT1 driven by maternal nutritional insults was reported to mediate PPARs and their target genes, thereby generating developmental programming of adult disease [84]. Maternal high-fat diet increased fetal fat mass accompanied by elevated PPARγ but decreased SIRT1 expression [85]. As we mentioned earlier, maternal high-fructose diet induced several phenotypes of metabolic syndrome in adult offspring [46]. Using the maternal high-fructose rat model, we found PPAR signaling pathway is significantly regulated in the offspring liver, heart, and kidney using next-generation RNA sequencing (NGS) analysis [48,86].

On the other hand, pharmacological interventions targeting AMPK or PPARs signaling have been reported to prevent the development of hypertension and metabolic syndrome in a variety of fetal programming models [84,87]. Although nutrient-sensing signaling are not likely to be the sole mechanism that increases susceptibility to disease in later life, it is required to elucidate their interplay with other mechanisms of nutritional programming in determining its impact on adult diseases of developmental origins.

### 5.2. Oxidative Stress

The embryo and fetus have low-antioxidant capacity. An overproduction of reactive oxygen species (ROS) under suboptimal intrauterine conditions would prevail over antioxidants, leading to oxidative stress damage and thus compromising fetal development [88]. These damages include oxidation of biological molecules, like lipids, proteins, and DNA. Excessive pro-oxidant molecules can overpower the antioxidant defense, resulting in biological damage, namely, oxidative stress. There are several types of nutritionally mediated oxidative stress sources, including diets high in carbohydrates, animal-based protein, and saturated fat [89]. Oxidative stress during pregnancy may lead to lifelong effects on vulnerable organs leading to adverse offspring outcomes in later life [90]. As reviewed elsewhere [91], several nutritional insults in pregnancy have been linked to oxidative stress in adult offspring, including maternal caloric restriction [33,35], high-fructose diet [48], low-protein diet [57], high-fat diet [64,65], zinc-deficiency diet [75], iron-deficiency diet [92,93], and methyl-donor diet [81]. Detailed mechanisms that underlie the interactions between maternal nutrition and oxidative stress and their roles in the programming process toward adult diseases of developmental origins, however, remain to be determined.

The major antioxidant nutrients in our body are copper, zinc, manganese, selenium, vitamins E, C, and A, and the glutathione system. Although some prospective assessment of the effect of supplemental antioxidants on human health suggests benefit, there are conflicting results in this area [94]. Although antioxidant nutrients therapy in pregnancy have been shown to prevent adult NCDs [33], there is a lack of data on when and how to use antioxidant nutrients to reprogram oxidative stress-related adult diseases.

### 5.3. Tissue Remodeling

In the first trimester, there is rapid differentiation and development of organs and systems. The second trimester is characterized by the development of structures and the start of functional activities. The third trimester is a period of the most rapid growth during the gestation. Most organ development lasts to birth, while some others continue to develop after birth, like the liver and nervous system [95].

If nutritional insults in gestation impact on critical periods of rapid growth, then it would be expected that the organs will mature at a smaller size and with a reduced functional capacity. For example, maternal low-protein diet caused fewer islets, smaller islets, and reduced islet vascularization in rat offspring pancreas [96]. The same maternal insult also influenced the thymic growth in a low-protein diet mice model [97].

Another example is the kidney. Low nephron number plays a key role in the developmental programming of cardiovascular disease, hypertension, and kidney disease [8]. There is an increasing body of literature demonstrating the relationship between maternal malnutrition and low nephron number, as reviewed elsewhere [98,99]. In rodent models, reported nutritional insults include maternal caloric restriction [35], low-protein diet [56], high-salt diet [69], low-salt diet [69], zinc deficiency [75], vitamin A restriction [100], and iron restriction [101]. In low-protein diet model, adult male offspring developed low nephron number and hypertension, which is related to renal hyperfiltration and activation of the renin–angiotensin system (RAS) [56]. On the other hand, nutritional programming can also have no effect [102], or even augment nephron number in adult offspring [103]. These observations highlight that the nutritional programming might be not driven by a single factor (i.e., low nephron number) and other organ- or phenotype-specific mechanisms remain to be identified.

### 5.4. Epigenetic Regulation

Persistent epigenetic modification of genes linked to disease is considered as a crucial mechanism for developmental programming [104]. Epigenetics is defined as alterations in gene expression that are not explained by changes in the DNA sequence. Epigenetic mechanisms include DNA methylation, histone modification, and non-coding RNAs, all of which are involved in translating nutritional insults in pregnancy to the developmental programming of adult diseases [104].

In rats, low-protein diet in pregnancy was shown to alter methylation and expression levels of *PPARα* gene and glucocorticoid receptor (GR) genes in the liver of the offspring [105]. Of note is that such alterations can be reversed by folic acid supplementation. Using DNA microarray technology, a previous study showed maternal low-protein diet caused a global upregulation of genes implicated in adipocyte differentiation, as well as in protein, carbohydrate, and lipid metabolism in the visceral adipose tissue [106]. Additionally, global methylation patterns in the offspring liver and brain have been studied in two different programming models, low-protein diet model [107], and micronutrient deficiency model [108], respectively. Several genes belong to the renin–angiotensin system have been reported to be altered by epigenetic regulation, culminating to the development of hypertension [109]. Using a mouse model, a report showed that maternal low-protein diet caused significant increases in the mRNA and protein expression of angiotensin converting enzyme-1 (ACE1) in offspring brain, with changes in DNA methylation and miRNA [110]. Furthermore, maternal undernutrition resulted in epigenetic changes in hypothalamic genes, GR, neuropeptide Y, and proopiomelanocortin, which predispose offspring to altered regulation of food intake and glucose homeostasis in later life [111].

Another important point to note is the impact of methyl-donor nutrients involved in the DNA methylation. It has been reported that maternal serum vitamin B12 levels inversely correlated with offspring’s global methylation status at birth [112]. Another report showed that folic acid supplementation directly impacted the methylation status of the IGF2 gene in the infants up to 17 months of age [113]. Moreover, maternal blood concentrations of methyl-donor nutrients measured around the time of conception can predict the methylation patterns at metastable epialleles in their offspring [114].

Overall, these studies support that malnutrition in pregnancy can epigenetically program development of adult diseases in later life. Nevertheless, the detailed mechanisms underlying the epigenetic modulation of common genes by different maternal nutritional insults still await for further study in diverse models of developmental programming.

### 5.5. Gut Microbiota

There is now growing evidence suggesting the role of early-life gut microbiota in developmental programming of metabolic syndrome-related disorders [115,116]. Maternal nutritional insults may cause a microbial imbalance, namely dysbiosis. Dysbiosis of gut microbiota may link early-life nutritional insults and later risk of adult diseases [117]. The gut microbiota produces a variety of metabolites detectable in host circulation, including short-chain fatty acids, small organic acids, bile acids, vitamins, and choline metabolites [118]. During pregnancy, the diet–gut microbiota interactions can mediate changes in global histone acetylation and methylation not only in mother but also in the fetus via the contact with their metabolites [119]. Emerging evidence shows that gut microbiota dysbiosis in early-life might be correlated with adverse offspring outcomes including obesity [120], metabolic syndrome [120], diabetes [120], hypertension [121], allergy [122], and neurological disorder [123]. On the other hand, non-essential nutrients are substances within foods that can have a significant impact on health, for example, dietary fiber. Of note, consumption of dietary fiber has become one dietary strategy for modulating the gut microbiota. A recent study from our laboratory examined the maternal high-fructose-induced hypertension model and found that modulation of gut microbiota by inulin is able to protect adult offspring against programmed hypertension [124]. Although recent studies demonstrating that microbiota-targeted therapies can be applied to a variety of diseases [125], their roles on nutritional programming-related disorders, especially the use of prebiotics in pregnancy, demand further exploration.

### 5.6. Sex Differences

There is a growing body of evidence that indicates sex differences exist in nutrient intake, bioavailability, and metabolism [126,127]. Several above-mentioned mechanisms related to nutritional programming, such as nutrient-sensing signal [128], oxidative stress [129], and epigenetic regulation [130] have been reported to respond to maternal nutritional insults in a sex-specific manner.

Same maternal nutritional insults, such as caloric restriction [34], high-fructose diet [49], or high-fat diet [67], can induce different phenotypes on male and female offspring. For example, male offspring are more prone to hypertension than female offspring [5]. This difference has led many studies to target their efforts totally to one sex, especially to males [35,56,57,69,75].

Evidence from animal models of maternal overnutrition has suggested that females are more vulnerable to programming of glucose homeostasis, whereas males are more susceptible to changes in adiposity and body weight [131,132,133]. On the other hand, male offspring of pregnant rats with caloric restriction during early gestation displayed obesity later in life, but female offspring were unaffected [134].

A few studies have investigated the sex-specific programming response to maternal diet on transcriptome profiles of the offspring [49,135]. A previous study has shown that more genes in the placenta were affected in females than in males under different maternal diets [135]. This is in accord with a report revealing that sex-specific placental adaptations are often associated with male offspring developing adult disease while females are minimally affected [136]. In a maternal high-fructose diet model [49], we found a sex-specific alteration of renal transcriptome response to maternal high-fructose intake and female offspring are more fructose-sensitive. However, whether higher sensitivity to nutritional insults is beneficial or harmful for programming effects in female offspring awaits further evaluation. As males and females likely respond differentially to nutritional programming, future research is required to clarify whether nutritional interventions also result in sex-specific reprograming effects.

## 6. Nutritional Interventions as Reprogramming Strategies

Reprogramming strategies aim to reverse the programmed development and achieve normal development. These strategies could be nutritional intervention, exercise, lifestyle modification, or pharmacological therapy. Nutritional programming is theoretically bidirectional. Inadequate (e.g., folate deficiency) or excessive intake (e.g., excess of macronutrients) of certain nutrients in pregnancy leads to adult diseases in later life. Conversely, adverse programmed processes during a compromised pregnancy can be prevented or at least reduced by appropriate nutritional interventions. It is well known that a balanced, diverse, and nutritious diet is universally recommended to meet nutritional needs to improve maternal and birth outcomes [137,138]; however, little is known whether supplementing with certain classes of nutrients in gestation can be beneficial on DOHaD-related disorders induced by various early-life insults in humans. In the current review, we only restrict to nutritional interventions during pregnancy and/or lactation periods as reprogramming strategies to prevent adult diseases of developmental origins in all sorts of animal models, some of which are listed in Table 2 [33,35,124,139,140,141,142,143,144,145,146,147,148,149,150]. Rats are the most commonly used species for developmental programming research. Rats become sexually mature at approximately 5–6 weeks of age. In adulthood, one rat month is comparable to three human years [151]. Accordingly, Table 2 lists the ages of reprogramming effects measured in rats ranging from 4 to 14 months, which can be translated to humans of specific age groups, from childhood to young adulthood. However, limited information is available about the long-term effects of nutritional interventions on older adult offspring. Additionally, developmental window is not uniform across different organ systems or species. For example, renal development in rodents, unlike in humans, continues up to postnatal week 1–2 [99]. Therefore, nutritional interventions during pregnancy and early lactation period may preserve nephrogenesis and increase nephron numbers to prevent the developmental programming of kidney disease [35,140].

Most importantly, some nutritional interventions are employed in non-nutritional-insults-induced programming models [140,141,142,144]. Nutritional interventions are grouped into macronutrients, micronutrients, and non-essential nutrients.

### 6.1. Macronutrieents

Macronutrients used as reprogramming strategies are mainly directed at amino acids. Within the body, amino acids are used for a wide variety of structural proteins and, hence, they play critical roles in organogenesis and fetal development. Among them, the most commonly used for reprogramming is citrulline. Maternal citrulline supplementation has been reported to protect adult offspring against hypertension and kidney disease in several models of developmental programming, including maternal caloric restriction [35], streptozotocin-induced diabetes [140], prenatal dexamethasone exposure [141], and maternal nitric oxide (NO) deficiency [142]. Citrulline is a precursor to arginine, and it is involved in the generation of NO [152]. Despite post-weaning arginine supplementation being reported to improve hypertension, insulin sensitivity, and beta cell function in offspring rats [153,154], the reprogramming effects of arginine supplementation in pregnancy are yet to be examined. Supplemental citrulline is more efficient than arginine to produce NO as citrulline can bypass hepatic metabolism and allow renal conversion to arginine. Thus, a better understanding of maternal citrulline supplementation in the prevention of diverse animal models of developmental programming is warranted before it is implemented in humans.

Another three amino acids, glycine, branched-chain amino acid, and taurine have also been used as reprogramming interventions in models of maternal low-protein diet [139], maternal caloric restriction [143], and streptozotocin-induced diabetes [144], respectively. Glycine is a simple amino acid not essential to the human diet. Glycine and vitamins (folic acid, vitamin B2, B6, and B12) take part in one-carbon metabolism and DNA methylation. Thus, glycine supplementation may have important implications for fetal programming through epigenetic mechanisms. Additionally, maternal branched-chain amino acid supplementation protects adult rat offspring against hypertension in a maternal caloric restriction model [143]. Taurine is an abundant semi-essential, sulfur-containing amino acid. Taurine supplementation during gestation prevented maternal diabetes-induced programmed hypertension [144]. Taurine supplementation can lower BP and increase hydrogen sulfide simultaneously [155]. Cumulative evidence supports hydrogen sulfide as a reprogramming strategy for long-term protection against programmed hypertension [156]. Additional studies are required to clarify whether the protective effects of maternal taurine supplementation on programmed hypertension is related to hydrogen sulfide pathway in other programming models.

So far, three reports showed maternal polyunsaturated fatty acids’ (PUFAs) supplementation has reprograming effects against hypertension [145,146], cardiac remodeling [146], and liver steatosis [147] in adult rat offspring. Although PUFAs have been recommended for pregnant and breastfeeding women [138], a recent meta-analysis that included 3644 children showed maternal supplementation with omega-3 PUFA during pregnancy does not have a beneficial effect on obesity risk [157]. Thus, whether there are differential reprogramming effects of individual PUFAs used as dietary supplement during pregnancy on diverse adult diseases remain to be determined.

### 6.2. Micronutrients

Micronutrients include vitamins and minerals. Relatively few reports demonstrated reprogramming effects of maternal micronutrients supplementation on offspring outcome [33,148,149]. One previous report demonstrated that combined micronutrients vitamin C, E, selenium, and folic acid supplementation in gestation prevented maternal caloric restriction-induced hypertension and endothelial dysfunction [33]. Vitamin C, E, and selenium have antioxidant properties. These micronutrients were shown to prevent programmed hypertension by reducing oxidative stress [158]. Folic acid is known to prevent neural tube defects, leading to global recommendations for folic acid supplementation before and during early pregnancy. Besides, folic acid, combined with other vitamins B12, B6, and B2, are the source of coenzymes which participate in one-carbon metabolism. In our body, one-carbon metabolites serve as methyl-donors that are required for DNA methylation. Although folic-acid supplementation during pregnancy was reported to improve offspring cardiovascular dysfunction induced by maternal low-protein diet [148], whether its reprogramming effects is related to epigenetic regulation remains undiscovered. On the other hand, our recent study showed that pregnant rats fed with high-methyl-donor diet or methyl-deficient diet both resulted in programmed hypertension in their male adult offspring [81]. Additional studies are required to approve whether methyl-donor food intake in pregnant women is beneficial to reduce adverse offspring and not just birth outcomes [80]. In a rabbit model, maternal vitamin E supplementation protected adult offspring against hypertension and hypercholesterolemia [135]. Many of the physiological functions of vitamin E, including its antioxidative effects, have been well studied. However, there still exists an ongoing debate regarding its beneficial effects on human health [159]. Despite recent studies demonstrating that antioxidant vitamins and minerals can be applied to a variety of diseases [94], their reprogramming effects on different adult diseases for dietary supplement use during pregnancy remain to be identified.

### 6.3. Non-Essential Nutrients

Dietary fiber, one of the non-essential nutrients, has been used as reprogramming strategies to prevent developmental programming of hypertension [124], hepatic steatosis, and insulin resistance [150]. Maternal supplementation with inulin protected adult rat offspring born to dams fed with high-fructose diet against the developmental programming of hypertension [124]. Another report demonstrated that maternal oligofructose supplementation attenuated hepatic steatosis and insulin resistance in adult rat offspring in a high-fat/-sucrose diet model [150]. Inulin and oligofructose are the most studied forms of prebiotic foods for beneficial gut microorganisms. As gut microbiota can influence development of sensitization and allergy, the World Allergy Organization (WAO) experts issued their guidelines on the use of prebiotics for all formula-fed infants in allergy prevention [160]. Nevertheless, whether prebiotics can be used in pregnant women for prevention of allergy in their offspring required further preclinical and clinical evidence.

Additionally, combined supplementations with high-fiber diet and acetate prevented hypertension in deoxycorticosterone acetate (DOCA)–salt hypertensive mice [161]. Acetate is the most abundant short-chain fatty acids, the major metabolites of gut microbiota. Considering that the gut microbiota dysbiosis is a key mechanism underlying nutritional programming, maternal supplementation with prebiotics or other microbiota-targeting nutritional interventions (e.g., probiotics and postbiotics) might be used as reprogramming strategies in different adult diseases of developmental origins.

It is noteworthy that reprogramming strategies can be created based on the above-mentioned mechanisms leading to a variety of adult diseases. For example, maternal inulin supplementation prevented adult rat offspring against high-fructose diet-induced programmed hypertension associated with nutrient-sensing signals [124]. The beneficial effects of vitamin C and E, selenium, and folic acid [33], citrulline [140,142], conjugated linoleic acid [145], and folic acid [148] on developmental programming of hypertension are relevant to the reduction of oxidative stress. Moreover, low nephron number was restored by citrulline supplementation in the maternal caloric restriction model [35], streptozotocin-induced diabetes model [140], and prenatal dexamethasone exposure model [141]. Thus, nutritional interventions in pregnancy may reprogram common mechanisms of both nutritional and non-nutritional in utero insults to prevent offspring against adult diseases of developmental origins. This effect requires more efforts to bridge gaps between basic animal research and clinical practice.

## 7. Conclusions

Almost all NCDs can originate in early-life. It stressed the importance of taking a DOHaD approach to prevent and control global burden of NCDs. All nutrients in pregnancy play a pivotal role in fetal growth and development. Without a doubt, maternal malnutrition is definitely bad for offspring health as it induces nutritional programming, leading to the developmental programming of numerous NCDs. Conversely, nutritional programming can also be advantageous. Through animal models, several nutritional interventions have been proven effective as a reprogramming strategy to prevent the development of various NCDs. Although there is a great opportunity that reprogramming strategy is good for offspring health, there is also an ugly side to it.

Given that the complexities of nutritional programming in pregnancy lead to both beneficial and deleterious effects in mother and offspring health, there remains a long road ahead to determine the “right” nutritional intervention for the “right” person (mother or offspring) at the “right” time, to prevent the DOHaD-related diseases. It is clear that better understanding of the type of nutrient, dose of supplement, and therapeutic duration for nutritional interventions are needed before patients could benefit from this reprogramming strategy. We expect that studies utilizing animal models of nutritional programming will provide the much-needed scientific basis for the development of ideal reprogramming strategies for clinical practice that will improve pregnancy and offspring outcomes in humans.

## Figures and Tables

**Table 1:** Effects of malnutrition during pregnancy on adverse offspring outcomes in human cohort studies.

Nutritional Risk Factors	Outcome	Offspring Number	Age at Measure (Year)	Country	Cohort Study
Undernutrition					
Undernutrition	Hypertension, impaired glucose tolerance, and overweight	1339	40	Nigeria	Biafran famine study [15]
Undernutrition	Coronary heart disease, hyperlipidemia, obesity, obstructive airways disease, and microalbuminuria	741	50	Netherlands	Dutch famine study [16]
Undernutrition	Overweight, type 2 diabetes, hyperglycemia, and metabolic syndrome	1029–8973	55	China	China great leap forward famine study [17]
Undernutrition	Elevation of blood pressure	359	59	Netherlands	Dutch famine study [18]
Low vitamin B12	Insulin resistance	653	6	India	PMNS [19]
Low vitamin B12	Impaired cognition function	118	9	India	PMNS [20]
Vitamin D deficiency	Elevation of blood pressure	1834	5–6	Netherlands	ABCD [21]
Vitamin D deficiency	Elevation of blood pressure	3525	9.9	United Kingdom	ALSPAC [22]
Overnutrition					
Free sugar intake	Atopy and asthma	8956	7–9	United Kingdom	ALSPAC [23]
High-protein, low-carbohydrate diet	Elevation of blood pressure	253	40	Scotland	Aberdeen maternity hospital study [24]
High-protein, low-carbohydrate diet	Elevation of blood pressure	626	30	Scotland	Motherwell study [25]
High-protein intake	Elevation of blood pressure	434	20	Denmark	DaFO88 [26]
High-fat diet	Obesity	965	20	Denmark	Aarhus birth cohort study [27]

Studies tabulated according to nutritional risk factor and age at measure. ABCD, Amsterdam born children and their development; ALSPAC, the Avon longitudinal study of parents and children; CHDS, child health and development studies; CPP, the collaborative perinatal project pregnancy cohort; DaFO88 = Danish fetal origins cohort; MUSP, Mater University study of pregnancy and its outcomes; PMNS, Pune maternal nutrition study.

**Table 2:** Reprogramming strategies aimed at nutritional interventions to prevent developmental programming of adult diseases in animal models.

Treatments	Animal Models	Species/ Gender	Period of Treatment	Reprogramming Effects	Age at Measure	Reference
Macronutrients						
Glycine	Maternal low-protein diet, 9%	Wistar/M	Pregnancy	Prevented hypertension	4 weeks	[139]
Citrulline	Maternal caloric restriction, 50%	SD/M	Pregnancy and lactation	Prevented reduced nephron numbr and kidney injury	12 weeks	[35]
Citrulline	Streptozotocin-induced diabetes	SD/M	Pregnancy and lactation	Prevented reduced nephron numbr and hypertension	12 weeks	[140]
Citrulline	Prenatal dexamethasone exposure	SD/M	Pregnancy and lactation	Prevented reduced nephron number and hypertension	12 weeks	[141]
Citrulline	Maternal nitric oxide deficiency	SD/M	Pregnancy and lactation	Prevented hypertension	12 weeks	[142]
Branched-chain amino acid	Maternal 70% caloric restriction	SD/M	Pregnancy	Prevented hypertension	16 weeks	[143]
Taurine	Streptozotocin-induced diabetes	Wistar/M + F	Pregnancy and lactation	Prevented hypertension, hyperglycemia, and dyslipidemia	16 weeks	[144]
Conjugated linoleic acid	Maternal high-fat diet	SD/M	Pregnancy and lactation	Prevented hypertension and endothelial dysfunction	18 weeks	[145]
Omega-3 polyunsaturated fatty acids	Maternal low-protein diet, 5%	Wistar/M + F	Pregnancy and lactation	Attenuated hypertension and cardiac remodeling	6 months	[146]
Omega-3 polyunsaturated fatty acids	Maternal caferteria diet	SD/M + F	Pregnancy and lactation	Prevented liver steatosis	14 months	[147]
Micronutrients						
Vitamin C, E, selenium and folic acid	Maternal 50% caloric restriction	Wistar/M + F	Pregnancy	Prevented hypertension and endothelial dysfunction	14–16 weeks	[33]
Folic acid	Maternal low-protein diet, 9%	Wistar/M	Pregnancy	Prevented hypertension and cardiovascular dysfunction	15 weeks	[148]
Vitamin E	Cholesterol-enriched diet	Rabbit	Pregnancy	Prevented hypertension and atherosclerosis	6 and 12 months	[149]
Non-essential nutrients						
Long-chain inulin	Maternal high-fructose diet	SD/M	Pregnancyand lactation	Prevented hypertension	12 weeks	[124]
Oligofructose	Maternal high-fat/-sucrose diet	SD/M	Pregnancyand lactation	Attenuated hepatic steatosis and insulin resistance	21 weeks	[150]

Studies tabulated according to types of treatments and age at measure. SD, Sprague–Dawley rat. M, male; F, female.
